# Use of Miniature Step Gauges to Assess the Performance of 3D Optical Scanners and to Evaluate the Accuracy of a Novel Additive Manufacture Process

**DOI:** 10.3390/s20030738

**Published:** 2020-01-29

**Authors:** Maria Grazia Guerra, Leonardo De Chiffre, Fulvio Lavecchia, Luigi Maria Galantucci

**Affiliations:** 1 Department of Mechanics, Mathematics and Management, Politecnico di Bari, Via Orabona 4, 70125 Bari, Italy; 2 Department of Mechanical Engineering, Technical University of Denmark, Produktionstorvet 425, DK-2800 Kgs. Lyngby, Denmark

**Keywords:** 3D optical scanner, performance verification, polymer, ceramic, step gauge, optical interaction, uncertainty, accuracy, depth of field, additive manufacture, medical implant

## Abstract

In this work, we show how miniature step gauges featuring unidirectional and bidirectional lengths can be used to assess the performance of 3D optical scanners as well as the accuracy of novel Additive Manufacturing (AM) processes. A miniature step gauge made of black polyphenylene sulfide (PPS) was used for the performance verification of three different optical scanners: a structured light scanner (SLS), a laser line scanner (LLS), and a photogrammetry-based scanner (PSSRT), having comparable resolutions and working volumes. Results have shown a good agreement between the involved scanners, with errors below 5 μm and expanded uncertainties below 10 μm. The step gauge geometry due to the bidirectional lengths, highlights that there is a different interaction between the optical properties of the step gauge under measurement and each optical instrument involved and this aspect has to be considered in the uncertainty budget. The same geometry, due to its great significance in the detection of systematic errors, was used, as a novelty, to evaluate the accuracy of Lithography-based Ceramics Manufacturing (LCM), a proprietary additive manufacturing technology used for the fabrication of medical implants. In particular, two miniature step gauges made of Tricalcium Phosphate (TCP) were produced. Measurements conducted with the SLS scanner were characterized by a negligible error and by an uncertainty of about 5 μm. Deviations of the manufactured step gauges with respect to the Computer Aided Designed (CAD) model were comprised between ±50 μm, with positive deviations in the order of 100 μm on vertical sides. Differences in the order of 50 μm between the two step gauges were registered.

## 1. Introduction

Quality assurance of manufactured parts is a paramount aspect in the industrial field and 3D optical scanning systems are increasingly adopted for dimensional verification. As the geometrical complexity of industrial components increases, the advantages of using these technologies increase as well. Their capability to acquire large amounts of points in a relatively short time, also in the presence of free-form shaped parts, makes these techniques particularly attractive, as fast, flexible, and holistic systems [1]. When the close range is considered, the mostly adopted optical techniques are laser scanners, structured light scanners, and photogrammetry. In [2,3], comparative studies among different non-contact digitization techniques were conducted comprising 3D optical measuring systems and CT scanners. Laser scanners are currently applied for in-process inspection of 3D geometries [4,5,6], for biomechanical applications [7], and in the small range they are often used as oral scanners [8]. Photogrammetry, as well, is usually applied from large to close range [9] and, recently, it has also been applied to small objects with sub-millimeter features [10]. Structured light scanners are adopted as a consolidated technique in industrial metrology, for tolerance verification and, more specifically, in analyses involving the 3D comparison with respect to the Computer Aided Designed CAD model, [11] for the measurement of castings and cores in the automotive industry [12], for the measurement of turbine blades [13], in the biomedical field [14,15], and in the small range for evaluating wear on cutting tools [16]. Particularly relevant for this kind of instruments is the fringe projection mode, for which there are many approaches and an exhaustive overview is reported in [17]. Results reported on that research put emphasis on the shifting approaches, which allow obtaining dense reconstructions and high accuracy.

From a traceability point of view, 3D optical scanners present several limitations, due to many sources of errors involved in the measuring process. Currently, performance evaluation of such systems refers to the VDI/VDE 2634 Part 2 [18] and Part 3 [19], involving the usage of spheres and gauge blocks made of steel or ceramics measured throughout the scanning volume. Some applications of these guidelines are reported in [20,21,22], however, these standards are better suited just for first acceptance tests because these geometries and materials are not well-representative of the more complex industrial cases, and results cannot become generally valid for other measuring tasks. Other reference objects, closer to the real cases, were developed and tested in [23,24,25,26]. In [27] a calibration procedure for a laser scanner was carried out, considering a specifically designed ball plate. One of the biggest limits of the optical-based techniques is their dependency on surface characteristics, because of the different interaction between the measuring system and the optical properties of different materials and colors of the workpiece [28]. This topic has become of fundamental importance, since the usage of polymeric materials has strongly increased in many manufacturing fields.

In this work, we show how miniature step gauges featuring unidirectional and bidirectional lengths can be used to assess the performance of 3D optical scanners as well as the accuracy of novel Additive Manufacturing (AM) processes. These two topics, traceability of optical based scanners and the manufacturing accuracy assessment of an additive manufacturing process, are of paramount importance in the context of the fourth industrial revolution and they are strictly related since very often, due to their many advantages, optical scanners are used for verifying parts additively manufactured. Within this context, this research is a preliminary work, which put in evidence some important advantages of using the step gauge geometry for conducting two different, but fundamental, analyses. This reference object, already successfully implemented for systematic errors correction in Computed Tomography (CT) scanning [29,30,31], allows identifying several effects characterizing 3D optical instruments, which are not observed when gauge blocks or spheres are measured. The object has a simple geometry, and a tactile coordinate measuring machine (CMM) can be used for its calibration with low uncertainty. A miniature step gauge made of polyphenylene sulfide (PPS), was used as a reference object for performance verification of three optical scanning systems: a structured light scanner (SLS), a laser line scanner (LLS), and a photogrammetry-based scanner (PSSRT), having comparable resolutions and working volumes. Furthermore, an application of the miniature step gauge geometry is described for the dimensional analysis of the process of Lithography-based Ceramics Manufacturing (LCM) [32], a proprietary Additive Manufacturing (AM) technology developed by Lithoz [33] used for the fabrication of medical implants. Here, 3D optical scanning performed on step gauges produced by LCM can be used to evaluate the accuracy of the manufacturing process. 

## 2. Materials and Methods 

In the following sections, a description of the miniature step gauge, the measuring equipment used and the measuring procedure are reported, together with the description of the method for the uncertainty assessment.

### 2.1. Step Gauge Artifact 

Step gauges are typically obtained by assembling gauge blocks or other simple elements made of steel or ceramic materials. With the purpose of achieving high surface cooperativeness for the verification of 3D optical scanners, a miniature step gauge was introduced for the first time by replication using a bisacryl material for dental applications (Luxabite) [29]. Polymers represent a large number of industrial components but present drawbacks, in terms of stability over time and machinability with sufficient accuracy and surface quality [30].

**Figure 1:**
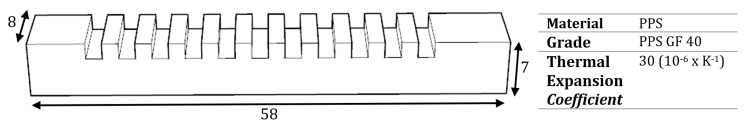
Step gauge: overall dimensions and material properties, dimensions are expressed in mm.

Miniature step gauges manufactured using different materials, such as aluminum, steel, and polymers, were successfully used for correcting systematic errors in Computed Tomography (CT) scanning [31]. The step gauge geometry is well suited for detecting and correcting systematic deviations since it features unidirectional as well as bidirectional lengths. The former are suitable for scale correction and they can be used for assessing the accuracy of a measuring system. Bidirectional lengths take into account the “probing” effect and they can be used for detecting effects due to the interaction between the measuring instrument and the optical properties of the workpiece. The overall geometry of the step gauge used in this work is shown in Figure 1, with overall dimensions of 58 × 8 × 7 mm^3^ and encompassing 11 grooves with 2 mm of depth and width. In connection with studies related to computed tomography, polyphenylene sulfide (PPS) with 40% of glass was found to be a good material for a step gauge, featuring low form errors, similar to those obtained using aluminum and steel, good thermal stability, low density, and good surface cooperativeness [30,31]. A black PPS miniature step gauge was, thus, used in this work. The surface roughness was also previously analyzed and it was found Ra = 0.82 μm and Rz = 6.19 μm. Measurements were carried out using a stylus profilometer Taylor- Hobson RTH Talysurf 5-120 with Z resolution of 0.001 μm.

### 2.2. Measuring Equipment and Scanning Parameters

Scanning parameters and strategies were defined for each instrument involved. Noise reduction was not applied in any case.

#### 2.2.1. Structured Light Scanner (SLS)

The structured light scanner, SLS, is a GOM ATOS III scan (GOM, Braunschweig, Germany), a blue light structured scanner with two cameras and a projector. In this investigation, the scanner was equipped with 90 mm lenses, which allow measuring a working volume of 60 × 45 × 30 mm^3^ with a resolution of 0.017 mm. The scanning strategy adopted is the following. With the aim to acquire the reference object entirely, the latter was positioned at the centre of a rotary table and a series of scans were carried out at every 22.5°, for a total number of 16 scans, while the sensor was tilted of 45° with respect to the rotary table (xy plane). Coded targets were used for the registration of the multi-view scans.

The exposure time, due to the black color of the step gauge was set to the maximum (1 sec), while the camera aperture was set and optimized during the calibration procedure, which was conducted with a calibrated pattern before measuring the two step gauges.

#### 2.2.2. Photogrammetry Based Scanner (PSSRT)

The photogrammetry based scanner used in this work is a Photogrammetric Scanning System with a Rotary Table (PSSRT) developed and placed at the Polytechnic University of Bari [34]. This is a flexible system with working volumes ranging from small volumes like 18 × 18 × 10 mm^3^ up to 150 × 150 × 40 mm^3^. The working volume strictly depends on the optical configuration chosen and, in this case, a Canon EOS 760D, a Canon EF 50 mm 1:1:8 II objective lens equipped with an extension tube of 20 mm, were used with a consequent basis resolution of 0.012 mm/pixel. The exposure time was set to 0.8 seconds and the f-stop was set to 20 in order to maximize the depth of field. The sensor was tilted 45°, and 72 pictures were taken rotating the object with steps of 5°. The scale error correction was carried out by means of the calibrated unidirectional lengths. Agisoft Photoscan v. 1.2.6 (Agisoft LLC, St. Petersburg, Russia) was used for the overall reconstruction process.

As an output of the analysis, the mesh was considered, since, for LLS and SLS, the meshing process is embedded in the scanning routine and it is not possible to export a point cloud. The reason for this choice is attributable to the easiness of managing a mesh rather than a point cloud [35]. On the other hand, errors due to the meshing approximation and simplification depend on the meshing algorithm adopted and on the number of the acquired points. In this case, the object surface was well-cooperative (high number of points acquired) and the error due to the meshing approximation was considered negligible. Moreover, it was empirically verified for the Photogrammetric Scanning System with a Rotary Table PSSRT and the meshing error was assessed by a comparison between the point cloud and the mesh obtained from that point cloud conducted with Cloud Compare free software (http://cloudcompare.org/) [36], using the point cloud-mesh comparison tool. As a result, it was much lower than 0.001 mm.

#### 2.2.3. Laser Based Scanner (LLS)

The laser scanner used was a 3SHAPE D800 (3Shape A/S, Copenhagen, Denmark), a laser line scanner, LLS, with a red beam (λ = 630–680 nm), with a tilting and rotating table and resolution of 0.02 mm. The scanning strategy adopted was a result from an optimization problem between the quality of results (minimum error with respect to the Coordinate Measuring Machine CMM values) and the scanning time. A tilt angle of 45° and a rotary stage of 36°, corresponding to 10 projections, were used.

### 2.3. Measurement Procedure

**Figure 2:**
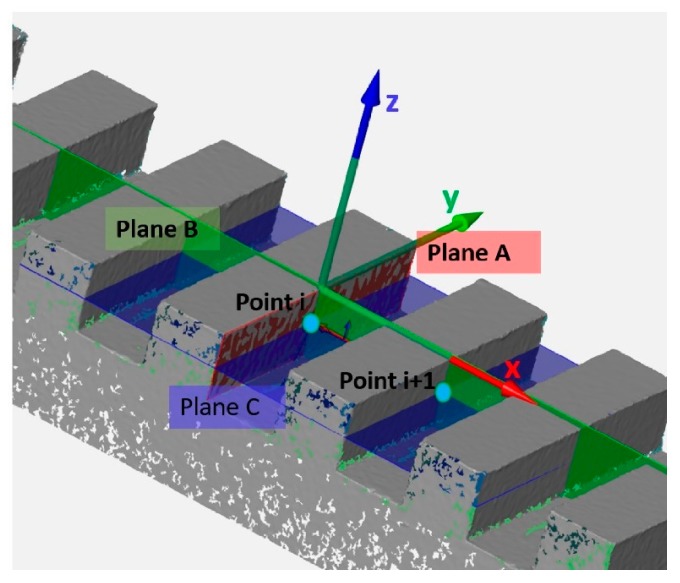
Definition of the 3-planes intersection points used for the lengths definition. The picture refers to a reconstructed mesh of the polyphenylene sulfide PPS step gauge.

The lengths were measured from groove side to groove side, as distances between two corresponding points, see Figure 2, representing the intersection between a plane fitted on each groove side, plane A, a symmetry plane (plane *xz*), Plane B, and, finally, a plane parallel to the xy plane and translated of −1 mm from the top, plane C.

**Figure 3:**
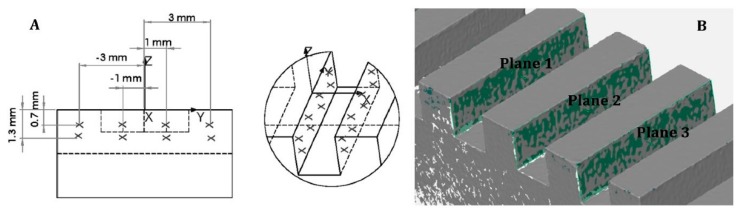
Coordinate measuring machine (CMM) probing strategy (**A**) and least square fitting procedure on acquired data with non-contact instruments (**B**).

The definition of the plane fitted on each groove side was carried out from eight points probed, for the Coordinate Measuring Machine (CMM), through the software Zeiss Calypso (https://www.zeiss.com/metrology/products/software/calypso-overview/calypso.html) [37], see Figure 3A, while the least square fitting method with a 3-sigma number of points was adopted for the optical instruments, as reported in Figure 3B, through the software GOM Inspect [38].

**Figure 4:**
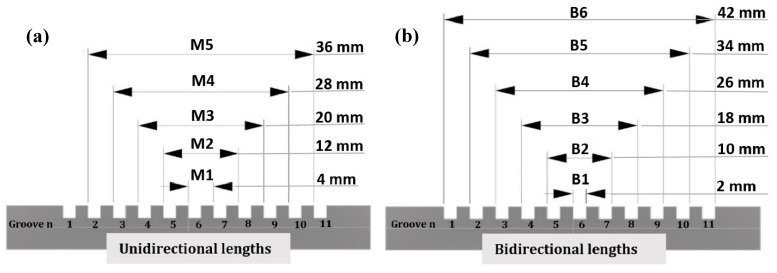
Unidirectional (**a**) and bidirectional (**b**) nominal lengths selected for the analysis.

The lengths were measured starting from the centre up to cover the entire length of the step gauge, which is, nominally 42 mm, for a total of five unidirectional lengths and six bidirectional lengths, see Figure 4. The lengths were numbered in ascending order, from M1 to M5 for the unidirectional lengths and from B1 to B6 for the bidirectional ones.

The step gauge was previously calibrated with a CMM Zeiss OMC 850 (Carl Zeiss AG, Oberkochen, Germany) equipped with a ø 0.8 mm probe, achieving an expanded uncertainty of 2 μm for the considered lengths.

### 2.4. Uncertainty Assessment

The uncertainty evaluation was conducted according to the ISO 14253-2 [39]. The uncertainty contributors are reported in Table 1 and the confidence level was set to 95%, corresponding to a coverage factor k equal to 2.

The general equation is reported in Equation (1).

(1) $$U=k*\sqrt{u_{r}^{2}+u_{w}^{2}\;+\;u_{e}^{2}+u_{p}^{2}}$$

**Figure 5:**
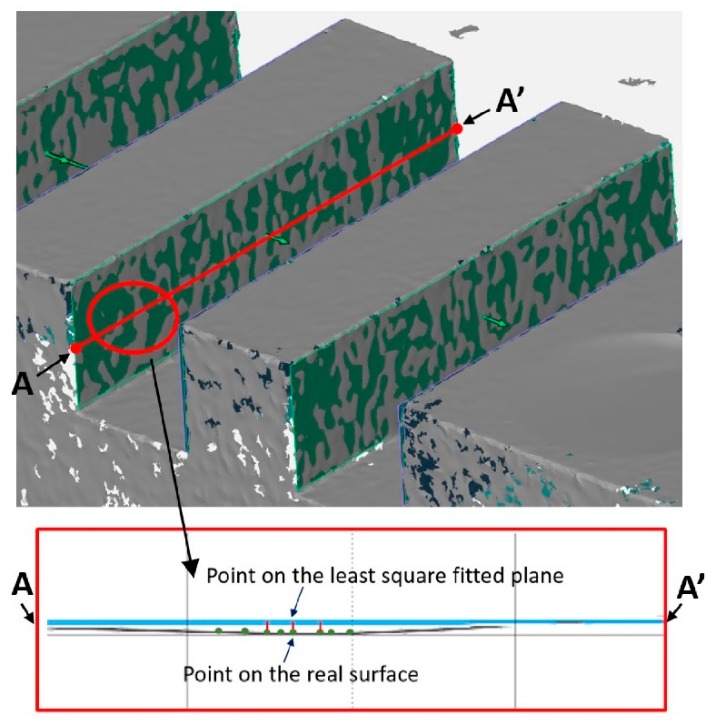
Fitting error description.

Starting from the general equation, the uncertainty of calibration was considered as the uncertainty of calibration of each single unidirectional and bidirectional length. The component due to the environmental temperature was also accounted for and the uncertainty due to the repeatability was computed considering five repetitions. The uncertainty coming from the workpiece was considered as the standard deviation of the fitting error, σfit, whose definition derives from the normal distances between points on the real surface and the corresponding points on the least square fitted plane, see Figure 5. This parameter was computed in the GOM Inspect software (https://gom.com/it/software-3d/gom-inspect.html) and describes the distribution of the points with respect to the fitted plane, the higher the variability of the points, the worse the reconstruction quality. The variability of the fitting error, σfit, was, then, chosen for considering the quality of the reconstructed surface, which is strongly affected by the optical interaction between the measurement system and the object surface.

**Figure 6:**
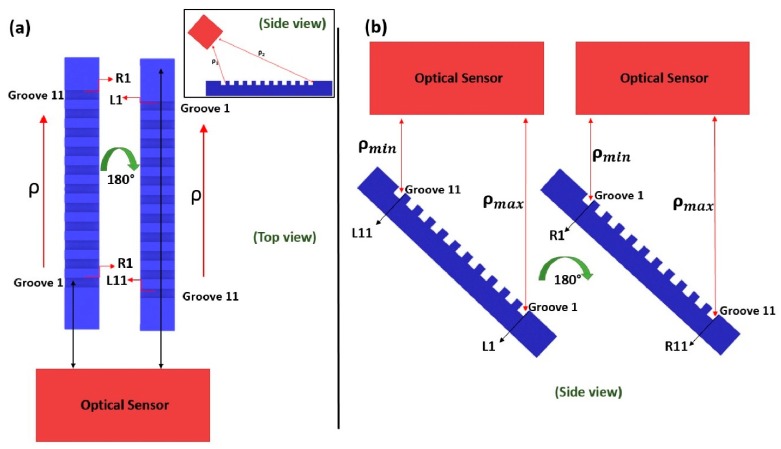
Distance from the sensor evaluated graphically for each groove side. (**a**) refers to the structured light scanner (SLS) and Photogrammetric Scanning System with a Rotary Table (PSSRT) and shows a top view, while (**b**) refers to the laser line scanner (LLS) and is a side view.

In particular, exploiting the step gauge geometry, the standard deviation of the fitting error, σfit, was analyzed, in relation to the distance from the sensor/light source (ρ), see Figure 6, considering the step gauge length, from the groove closest to the optical sensor, to the farthest groove. In Figure 6, considering the scanning strategy adopted for each scanner involved, the distances between the sensor and each groove side was graphically explained.

As it is possible to observe from Figure 6a, the one related to the Structured Light Scanner (SLS) and the Photogrammetric Scanning System with a Rotary Table (PSSRT), assuming just one focusing distance, the sensor inclined of 45° with respect to the rotary table plane and the scanning strategy involving just the rotation of the step gauge around the z axis, due to the particular configuration of the step gauge, there are fixed distances between the groove sides and the optical sensor, e.g., the groove side L1 is always the farthest, L11 the closest, and, as well, R1 the closest and R11 the farthest. It is the same for the LLS scanner, where the table, instead of the sensor, is inclined to 45° and the side view of the scanning strategy is the one shown in Figure 6b. Thus, the quality of reconstruction, indicated by the σfit parameter, could be related to the distance between the considered groove side and the optical sensor.

**Figure 7:**
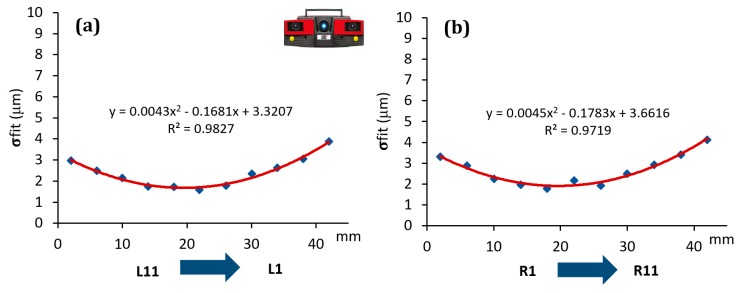
Trend of the sigma fitting error (σfit) evaluated considering the distance from the sensor for SLS scanner. Left groove sides (**a**) and right groove sides (**b**).

**Figure 8:**
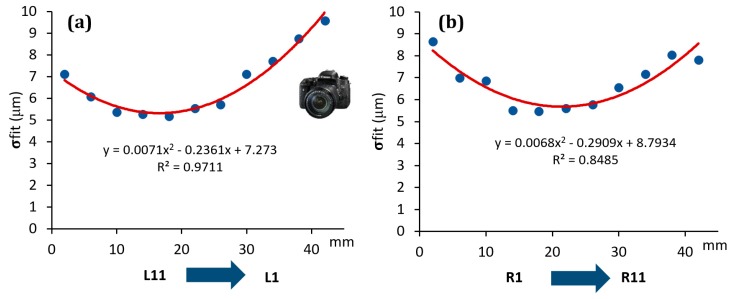
Trend of the sigma fitting error (σfit) evaluated considering the distance from the sensor for PSSRT scanner. Left groove sides (**a**) and right groove sides (**b**).

**Figure 9:**
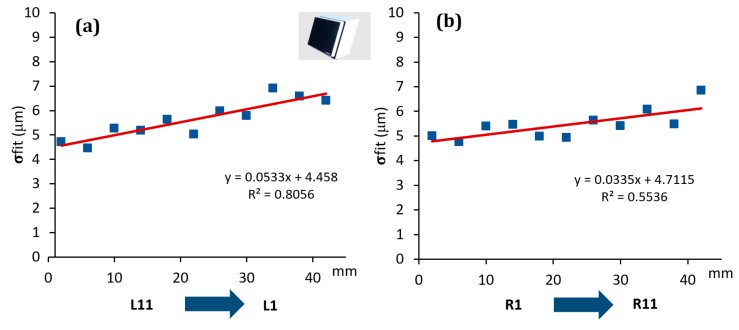
Trend of the sigma fitting error (σfit) evaluated considering the distance from the sensor/light source for LLS scanner. Left groove sides (**a**) and right groove sides (**b**).

The effect was, indeed, quantitatively analyzed for each scanner involved in the investigation, see Figure 7, Figure 8 and Figure 9. On the y-axis, the σfit was reported, while on the x-axis the groove sides ordered with respect to the distance from the optical sensor/light source (for simplicity this distance was considered ranging from the minimum value of 2 mm to the maximum of 42 mm, which is the maximum step gauge length).

**Figure 10:**
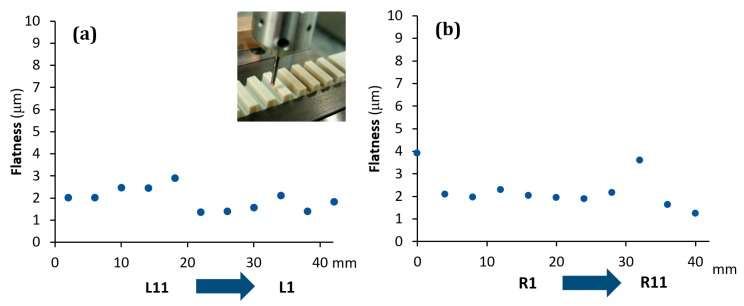
Flatness evaluated with the CMM. Left groove sides (**a**) and right groove sides (**b**).

As it is possible to observe, all the scanners showed a systematic effect. In particular, SLS showed a trend well fitted by a second order polynomial function, with its minimum corresponding approximately to the centre of the step gauge. The same function was found to well fit the data coming from PSSRT. A different trend was instead found for the Laser Line Scanner (LLS), where there is a linear trend and, as the distance from the sensor/light source increases the standard deviation of the fitting error increases as well, thus, quality of reconstruction decreases. It is important to underline that these effects, emerged during the analysis, are not due to the form errors of the step gauge, since the latter, calibrated with a CMM, was manufactured with a flatness, evaluated for each groove side, comprised in the range between 1 and 4 μm. Moreover, no trends were detected, and flatness values were randomly distributed throughout the step gauge length, as it was possible to observe in Figure 10.

All these effects could be attributable to different causes. For PSSRT, the depth of field is a criticality, since a very small working volume and a good resolution were achieved at the expense of the depth of field. This could also explain the shape of the trend registered, with the minimum at the centre of the step gauge, which is also the focusing point. Regarding the SLS, showing the same trend but within a smaller range (within 5 μm for the SLS and within 10 μm for PSSRT), it is possible to consider two different factors, the inhomogeneity of the light source within the working area, combined with the depth of field limitation. In this case, due to the black color of the step gauge, the exposition time used was the maximum value, 1 second. The best illuminated area was the one in the centre of the step gauge, while the worst was the one farthest from the sensor. Vertical sides placed very close to the sensor and the light source were also affected by a greater fitting error with respect to the central zone, and it could be due to the over exposition of that area. The first factor, related to the light source, is the most relevant, since in new experiment, conducted with the same scanner but with a more powerful light source, this phenomenon disappeared. The LLS was instead characterized by a fitting error increasing with the distance from the sensor and the laser source.

The highlighted effects are generalizable as effects due the optical interactions between the scanner and the object (depth of field, light source, and sensor distance) and their result can be accounted in the uncertainty budget only if a parameter like the sigma fitting error is considered. In the following analysis, errors evaluated with respect to the CMM values and uncertainties assessed according to Equation (1) were computed.

## 3. Results and Discussion

**Figure 11:**
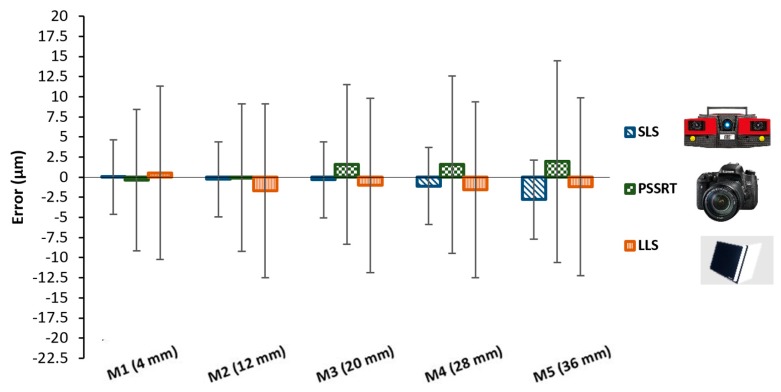
Results obtained considering unidirectional lengths.

**Figure 12:**
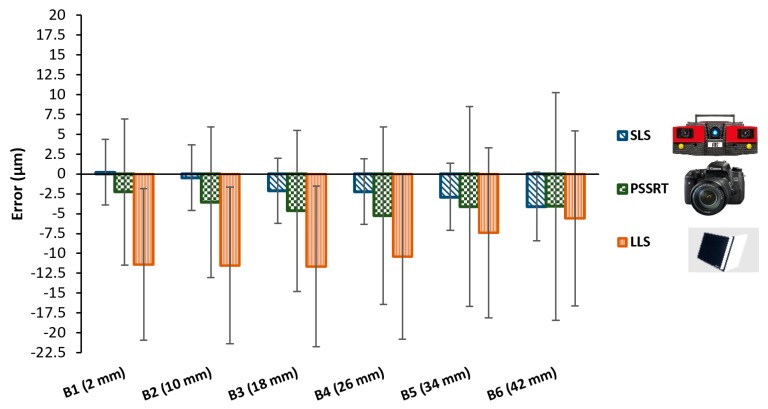
Results obtained considering bidirectional lengths.

Results of the comparison between each optical instrument and the calibrated measures are reported in the following graphs, see Figure 11 and Figure 12.

Considering the unidirectional lengths, see Figure 11, all the three optical scanners showed errors with respect to the Coordinate Measuring Machine CMM in the range within ±2.5 μm, with very small differences and uncertainties up to 10 μm. Uncertainties increased from the M1 (4 mm) to the M5 (36 mm). Bidirectional lengths, see Figure 12, highlighted more interesting differences, and they were, for definition, higher than errors registered for the unidirectional lengths. SLS still resulted in having measured values very close to the CMM values, with errors ranging from 0 to 4 μm and uncertainties within 10 μm. 

**Figure 13:**
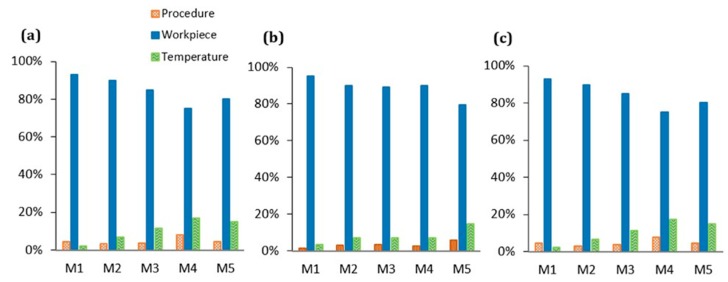
Percentage of each uncertainty component respect to the total uncertainty for the SLS (**a**), PSSRT, (**b**) and LLS (**c**), considering the unidirectional lengths.

Photogrammetric Scanning System with a Rotary Table (PSSRT) and the Laser Line Scanner (LLS) showed higher errors and uncertainties. The former, PSSRT, showed errors comprised between 4 and 9 μm and uncertainties up to 12 μm and increasing with the measurand length. The latter, LLS, showed errors comprised within 10 μm, with uncertainties up to 11 μm, increasing, as well, with the measurand length. In particular, the uncertainties registered for each scanner were strongly influenced by the workpiece related component, representing more than the 80% of the total expanded uncertainty, see Figure 13. Moreover, the increase of the uncertainty with the measurand length is justified by the measurand definition and the trends registered for the sigma fitting error along the step gauge length and explained in Figure 7, Figure 8 and Figure 9, as well as by the increasing of the component related to the thermal expansion.

## 4. Application to Additive Manufactured TCP Step Gauges

**Figure 14:**
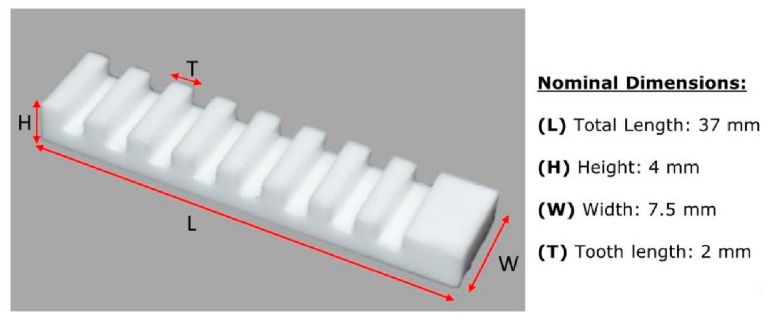
TCP miniature step gauge manufactured by Lithoz.

The importance of using 3D scanning systems is also related to the amount of information that those systems can yield [1]. A 3D optical based scanner is able to provide information for a 2D dimensional analysis, as well as, a 3D analysis involving a 3D comparison, which allows detecting, more immediately, the criticalities of a manufacturing process. In this case study, the Structured Light Scanner, SLS, described in Section 2.2.1, was selected for verifying the accuracy of a novel additive manufacturing process because it was found to be the most reliable scanner among the ones analyzed and the one showing the least optical interaction with the material of the step gauge. The step gauge geometry was used as a reference object (Figure 14) for the dimensional verification of the Lithography-based Ceramics Manufacturing (LCM) [30], a proprietary additive manufacturing (AM) technology developed by Lithoz, and used for the fabrication of medical implants. Two miniature step gauges made of Tricalcium Phosphate (TCP), a composite used in tissue engineering and maxillofacial surgery, were realized.

**Figure 15:**
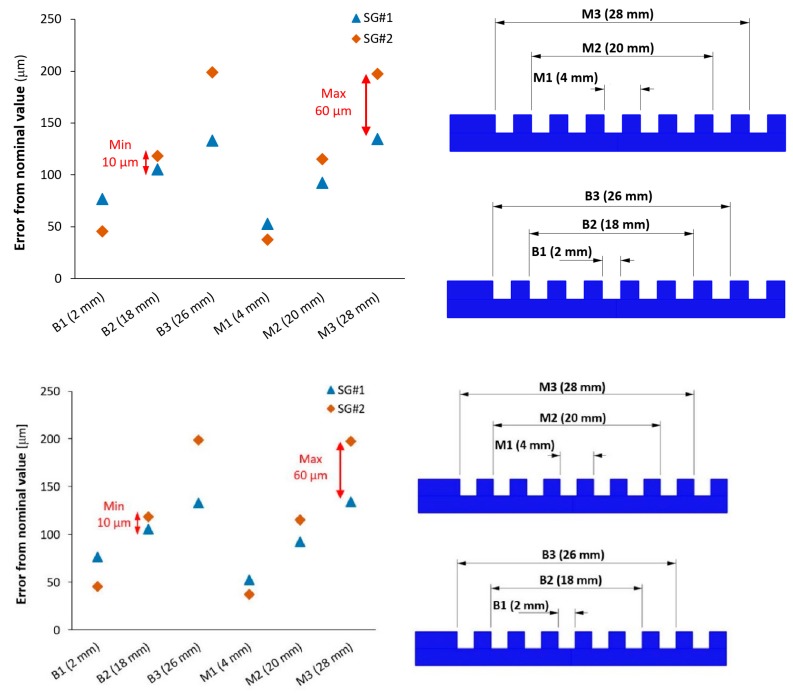
Differences between the two step gauges considering errors with respect to the Computer Aided Designed (CAD) dimensions.

The two step gauges belong to the same series and were manufactured with the same process parameters. In the following section, they will be indicated as SG#1 and SG#2. For the scanner acquisition, the same optical configuration reported in Section 2.2.1 was used, while the exposure time was changed due to the different color (white) of the step gauge, which required less light. The temperature during the acquisition was 20 ± 1 °C and the humidity was 49% ± 1%. The nominal dimensions and the measurands chosen for the dimensional analysis are reported in Figure 15.

Results, in terms of error with respect to the Computer Aided Designed (CAD) model, were in the order of 100 μm from the set dimensions, and SG#1 was more accurate, compared to SG#2, especially considering the maximum lengths M3 (28 mm) and B3 (26 mm). In Figure 15, the differences between the two step gauges are reported and they vary from 10 to 60 μm. The repeatability of the measurements, evaluated as a standard deviation of five repetitions, was 2 μm.

**Figure 16:**
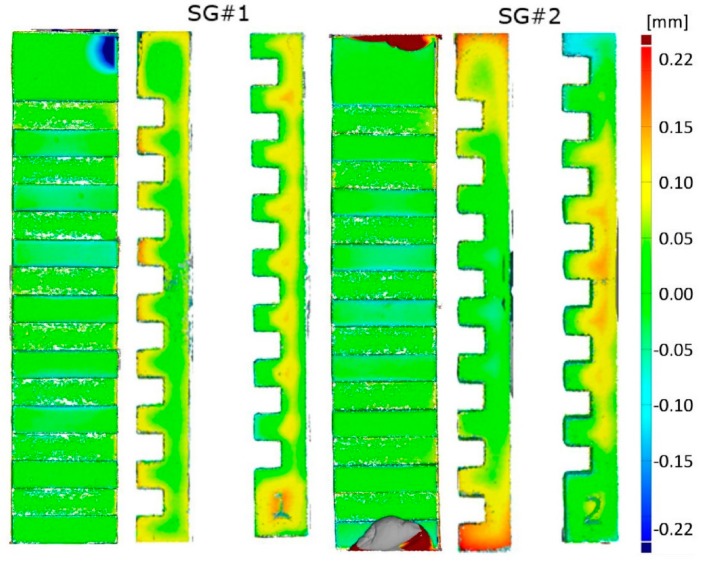
3D distance color map comparisons between the acquired meshes and the CAD model. Different colors, as reported in the colored map, indicate the range of deviations.

The use of a 3D scanner, with respect to the use of a contact Coordinate Measuring Machine (CMM), allows us to extend the analysis to the overall reconstruction. It is of paramount importance for the verification of the manufacturing process to understand where the deviations are located, in order to better point out the root cause of the fault. In this case, it was possible to observe some of the areas mostly affected by the manufacturing process, which can aid the manufacturer to understand the main criticalities, see Figure 16. Most of the points were comprised between ±50 μm of the deviation with respect to the CAD, while positive deviations in the order of 100 μm and they were registered on vertical sides. From the 3D comparison between the two step gauges, differences in the order of 50 μm were registered.

Finally, it is possible to associate to the measurement conducted on the ceramic step gauge an error and an uncertainty value coming from the values obtained in the previous sections. SLS was found to be the most reliable scanner, in terms of both, errors and uncertainty registered and, since the former were in the order of a few micrometers, much lower than the order of magnitude of the manufacturing errors, the error (bias) due to the instrument was considered negligible for the measurements on the Tricalcium Phosphate (TCP) step gauge, while for the uncertainty more attention must be paid. The latter was in the order of 5 μm for the PPS step gauge. However, in order to consider the different optical interactions, due to the different optical properties of the material, as well as, the different surface finish, the evaluation of the fitting error and its variability was analyzed and, in this case, no trend was detected. Considering each uncertainty component, following the Equation (1), only two were accounted: the one coming from the workpiece, evaluated as the average value of the σfit, and the one coming from the repeatability. The former evaluated on each groove side was about 7 μm, higher than the one registered for the polyphenylene sulfide PPS (about from 2 to 4 μm) and so was the second, since the repeatability over five repetitions was 2 μm (against the 0.5 μm of the PPS step gauge). The uncertainty component due to the temperature was considered negligible due to the low thermal expansion coefficient (about 10 × 10-6 k-1 of the TCP against the 30 × 10-6 k-1 of the PPS) and finally the uncertainty of calibration was not considered, since the SLS scanner was the reference instrument in this case study. Finally, it is possible to assess that the measurements conducted with the SLS scanner were affected by a negligible error and by an uncertainty of about 5 μm, which makes the SLS a powerful and reliable scanner for novel manufacturing processes verification, as in the case of TCP parts produced by Lithography-based Ceramics Manufacturing LCM.

## 5. Conclusions

In this work a polymer miniature step gauge, a reference object featuring unidirectional and bidirectional lengths, was used for the performance verification of three optical based scanners, a structured light scanner (SLS), a laser line scanner (LLS), and photogrammetry-based scanner (PSSRT), usually adopted for the reconstruction of very complex surfaces and applied in many research fields. The main finding was that all the scanners involved showed to be in very good agreement with the Coordinate Measuring Machine (CMM) calibrated values: errors registered on unidirectional lengths were within ±2 μm with uncertainties up to 10 μm for all the instruments involved. SLS still resulted to have measured values very close to the CMM values, with errors below 4 μm and uncertainties less than 10 μm. PSSRT showed errors comprised between 4 and 9 μm and uncertainties up to 12 μm, increasing with the measurand length. LLS showed errors comprised within 10 μm, with uncertainties up to 11 μm, as well increasing with the measurand length. The difference registered when comparing errors on bidirectional lengths, highlights that there is a different interaction between the optical properties of the step gauge under measurement and each optical instrument involved. This aspect was analyzed considering the standard deviation of the fitting error of each least square fitted plane. Results highlighted a different behavior of the scanners according to the different measurement principles, which was evaluated with respect to the sensor/light source distance from the object. The effect was taken into account in the uncertainty budget: uncertainty values were strongly affected by the above mentioned component related to the workpiece. Optical interactions are worth having further investigations, considering different materials and colors, as well as different optical measurement instruments.

In a further application, the SLS was used for the verification of a novel additive manufacturing process, LCM, used for fabricating medical implants with ceramic composites materials. The step gauge geometry was used as test geometry and the 3D models retrieved by the use of the scanner allowed us to obtain a full analysis, showing that the step gauges were manufactured with 95% of the surfaces lying in the range of ±100 μm deviation from the Computer Aided Designed (CAD) file, with an absolute average value of 50 μm. As a general conclusion, we have shown how miniature step gauges featuring unidirectional and bidirectional lengths can be used to assess the performance of 3D optical scanners as well as the accuracy of novel Additive Manufacturing (AM) processes. In particular, the step gauge geometry has put in evidence effects due to the optical interaction between a scanner and the object, which must be considered in the measurement uncertainty budget.

## Figures and Tables

**Table 1:** Uncertainty budget composition.

Uncertainty Component	Symbol	Type	Estimation	Distribution	SLS	LLS	PPSRT
Reference	*u_r_*	B	Reference Uncertainty	Rect.	*U*i/$\sqrt{3}$	*U*i/$\sqrt{3}$	*U*i/$\sqrt{3}$
					With i representing each measurand		
Optical Interaction Scanner/Workpiece	*u_w_*	A	Fitting Error	Rect.	σfitting error		
Environment	*u_e_*	B	Temperature variation	U-shaped	±1° C	±1.5° C	±1° C
Procedure	*u_p_*	A	Repeated measurements	Normal	σ_SLS/_$\sqrt{n}$	σ_LLS_$\sqrt{n}$	σ_PSSRT_$\sqrt{n}$
					With σ the standard deviation of n repetitions		
